# Supplementary material for: Efficient capture and storage of ammonia in robust aluminium-based metal-organic frameworks
Source: Commun Chem. 2023 Mar 24;6:55. doi: 10.1038/s42004-023-00850-4 (PMC10039057; doi:10.1038/s42004-023-00850-4)
Supplement: Supplementary file 2 — Supplementary Information [file 42004_2023_850_MOESM2_ESM.pdf]

Supplementary Information

Efficient Capture and Storage of Ammonia in Robust Aluminium-based Metal-Organic Frameworks

Lixia Guo,[1] Joseph Hurd,[2] Meng He,[1] Wanpeng Lu,[1] Jiangnan Li,[1] Danielle Crawshaw,[1] Mengtian Fan,[1] Sergei Sapchenko,[1] Yinlin Chen,[1] Xiangdi Zeng,[1] Meredydd Kippax-Jones,[1,3] Wenyuan Huang,[1] Zhaodong Zhu,[1] Pascal Manuel,[4] Mark D. Frogley,[3] Daniel Lee,[2] Martin Schröder[1]* and Sihai Yang[1]*

1. Department of Chemistry, University of Manchester, Manchester, M13 9PL (UK)
   M.Schroder@manchester.ac.uk; Sihai.Yang@manchester.ac.uk
2. Department of Chemical Engineering, University of Manchester, Manchester, M13 9PL (UK)
3. Diamond Light Source, Harwell Science and Innovation Campus, Oxfordshire, OX11 0DE (UK)
4. ISIS Facility, STFC Rutherford Appleton Laboratory, Chilton, Oxfordshire, OX11 0QX (UK)

Table of Contents

Supplementary Figures.......... S3

Supplementary Notes.......... S18

Supplementary Tables.......... S19

Supplementary References.......... S25

Supplementary Figures

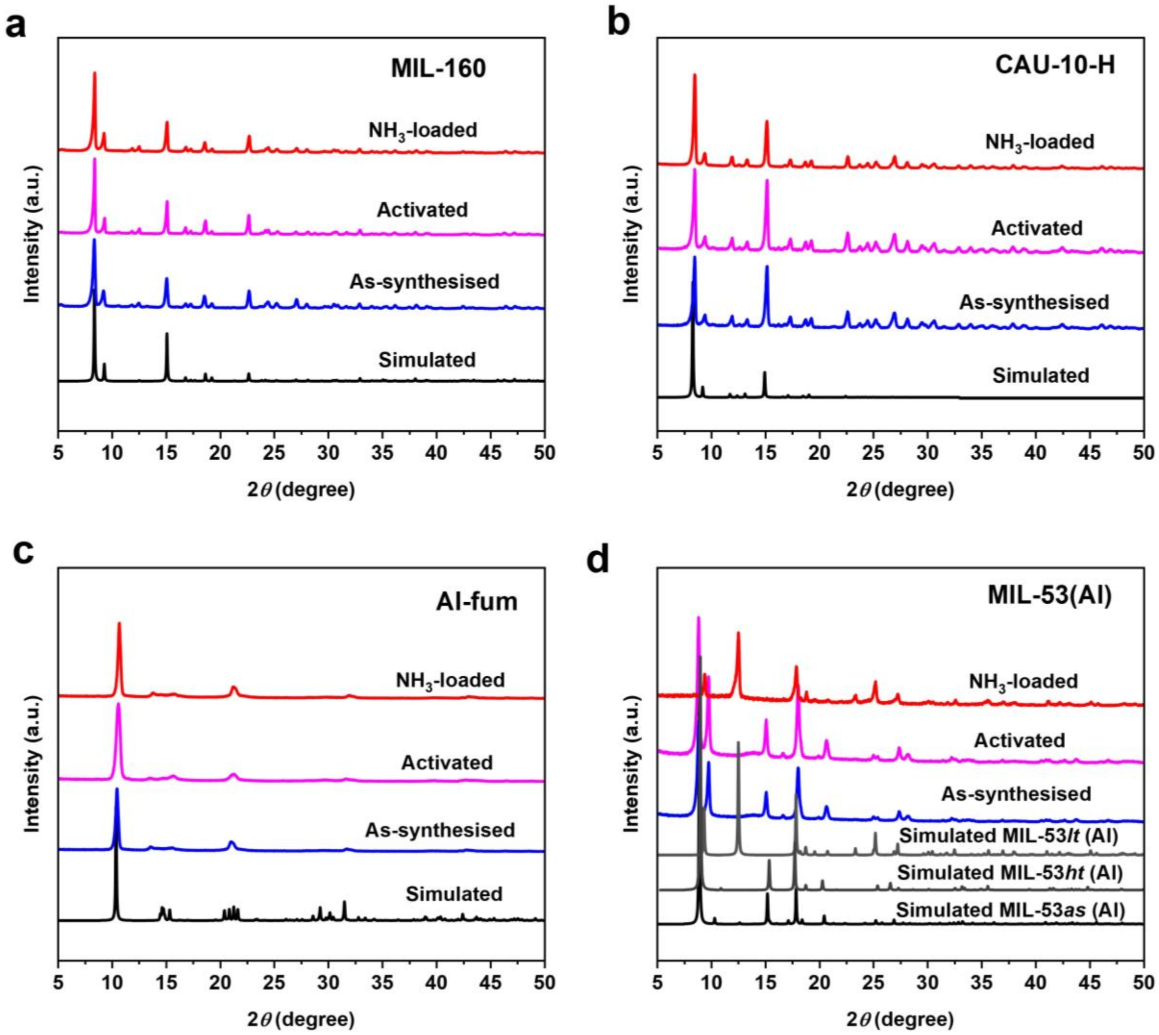

Supplementary Figure 1. Powder X-ray diffraction. PXRD patterns of simulated (black), as-synthesised (blue), activated (magenta) and NH_3-loaded (red) samples of (a) MIL-160, (b) CAU-10-H, (c) Al-fum and (d) MIL-53(Al).[1] MIL-53*as* (Al) is the form occupied by free terephthalic acid ligand; MIL-53*ht* (Al) is the calcined form with empty channels; MIL-53*lt* (Al) is the room temperature form with water molecule in the channels.

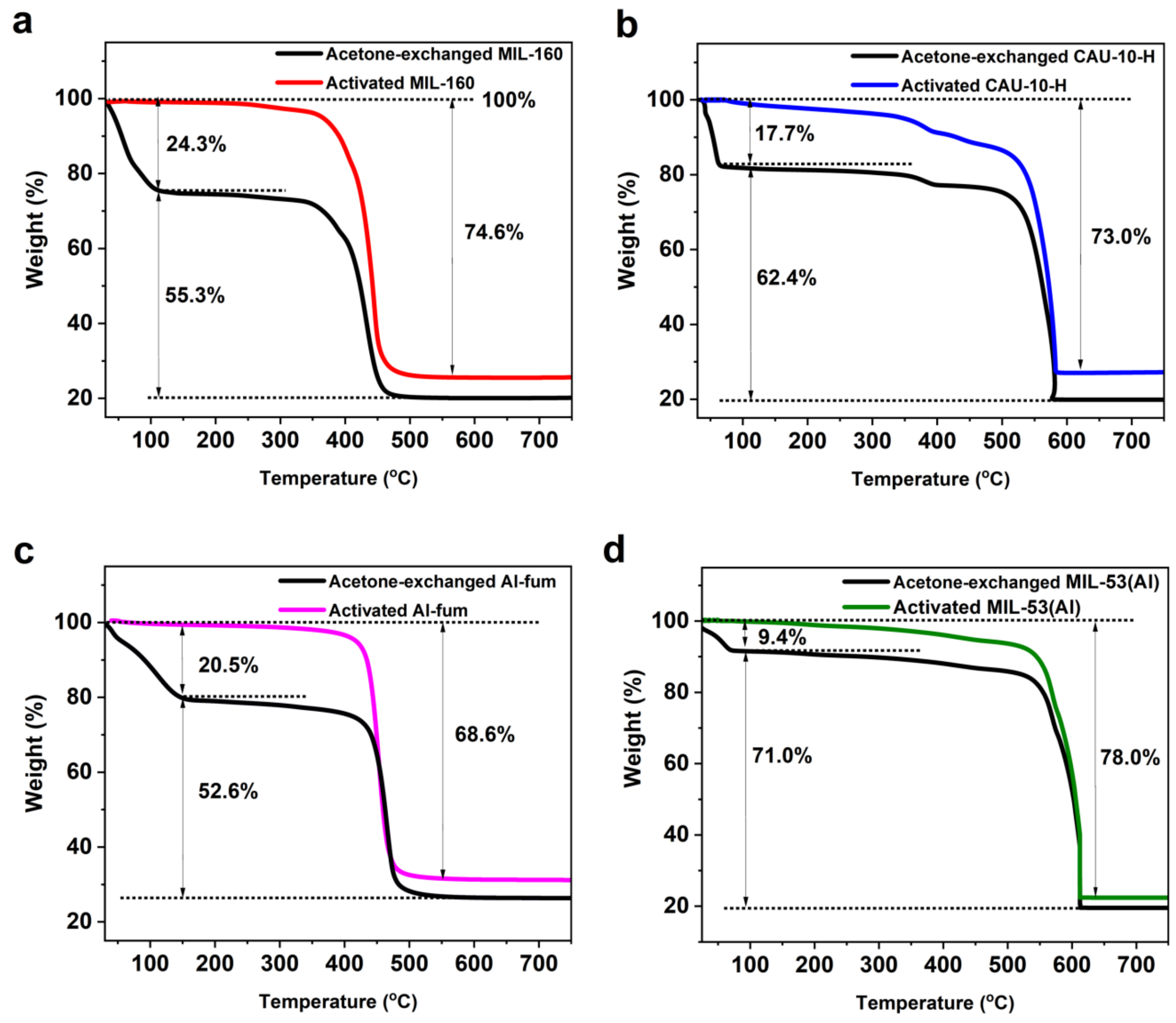

Supplementary Figure 2. Thermogravimetric analysis. TGA curves of acetone-exchanged (black) and activated samples for (a) MIL-160 (red), (b) CAU-10-H(blue), (c) Al-fum(magenta) and (d) MIL-53(Al) (olive) measured under an air flow.

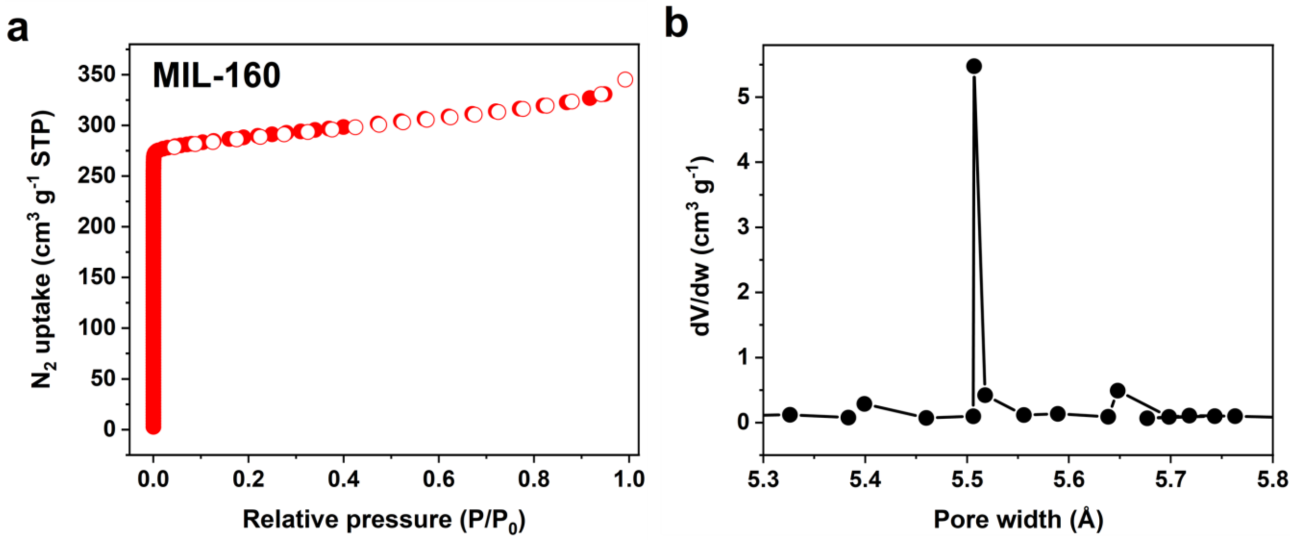

Supplementary Figure 3. Characterisation of porosity. (a) N_2 adsorption and desorption isotherms at 77 K and (b) micropore size distribution for MIL-160.

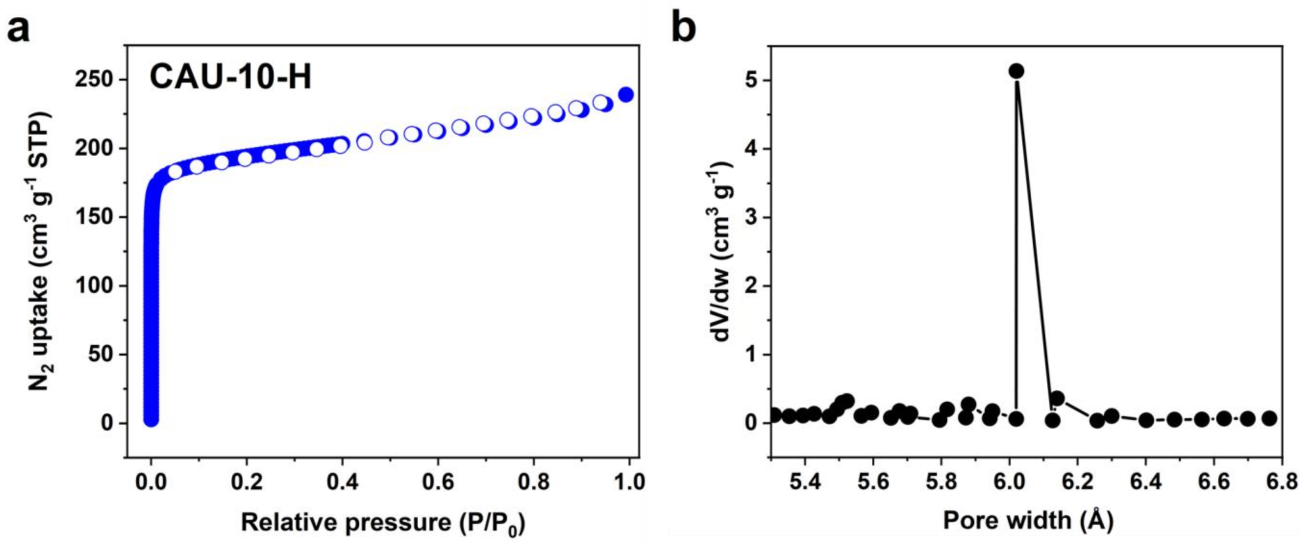

Supplementary Figure 4. Characterisation of porosity. (a) N_2 adsorption and desorption isotherms at 77 K and (b) micropore size distribution for CAU-10-H.

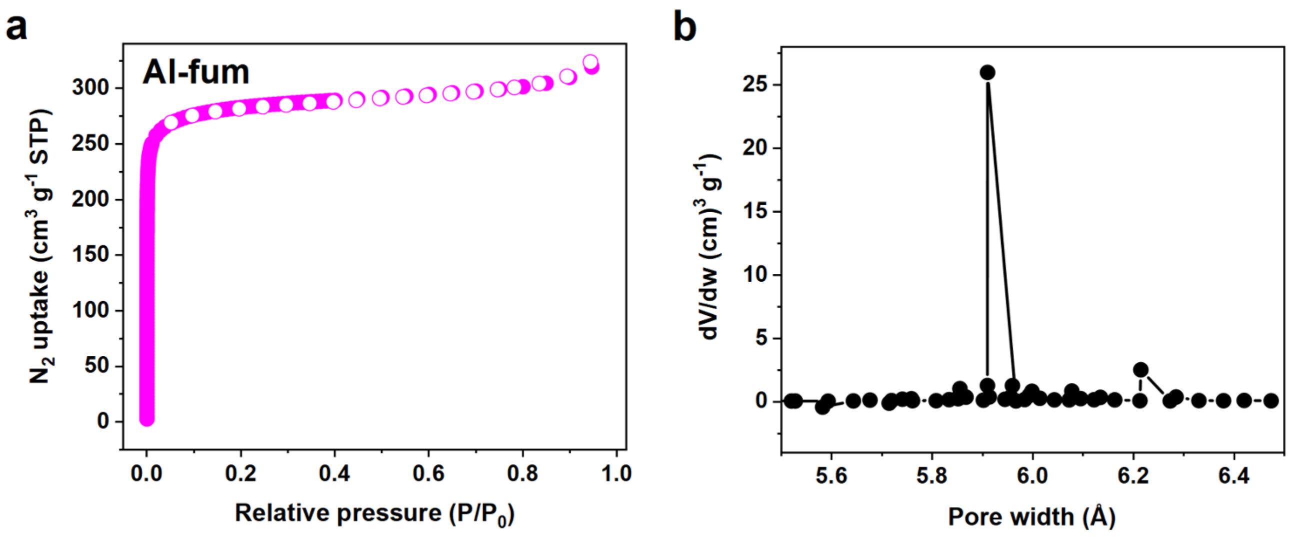

Supplementary Figure 5. Characterisation of porosity. (a) N_2 adsorption and desorption isotherms at 77 K and (b) micropore size distribution for Al-fum.

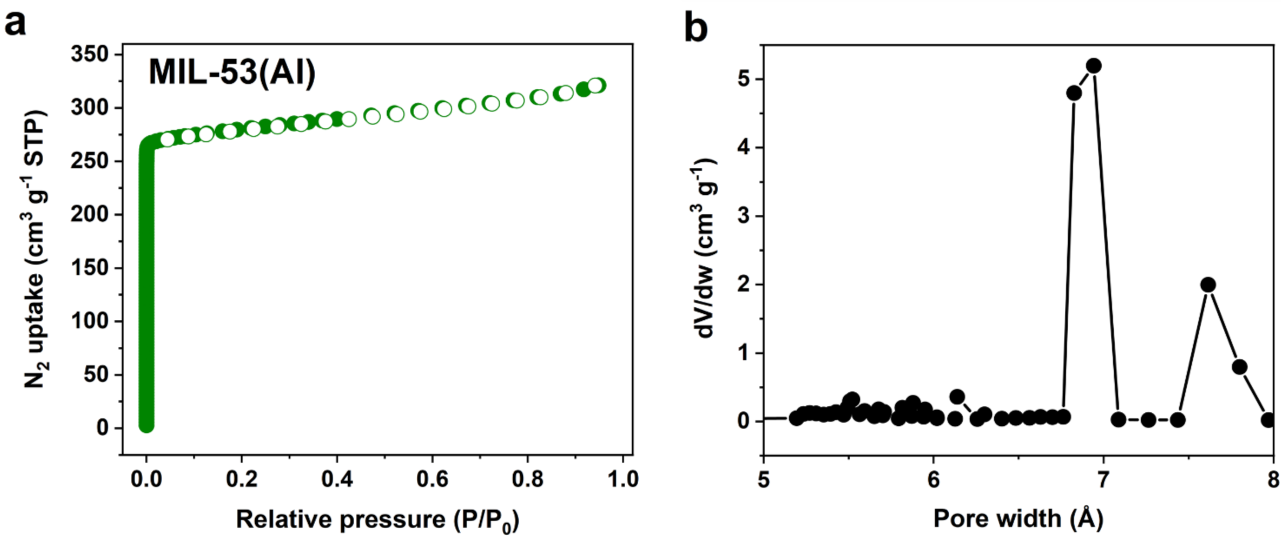

Supplementary Figure 6. Characterisation of porosity. (a) N_2 adsorption and desorption isotherms at 77 K and (b) micropore size distribution for MIL-53(Al).

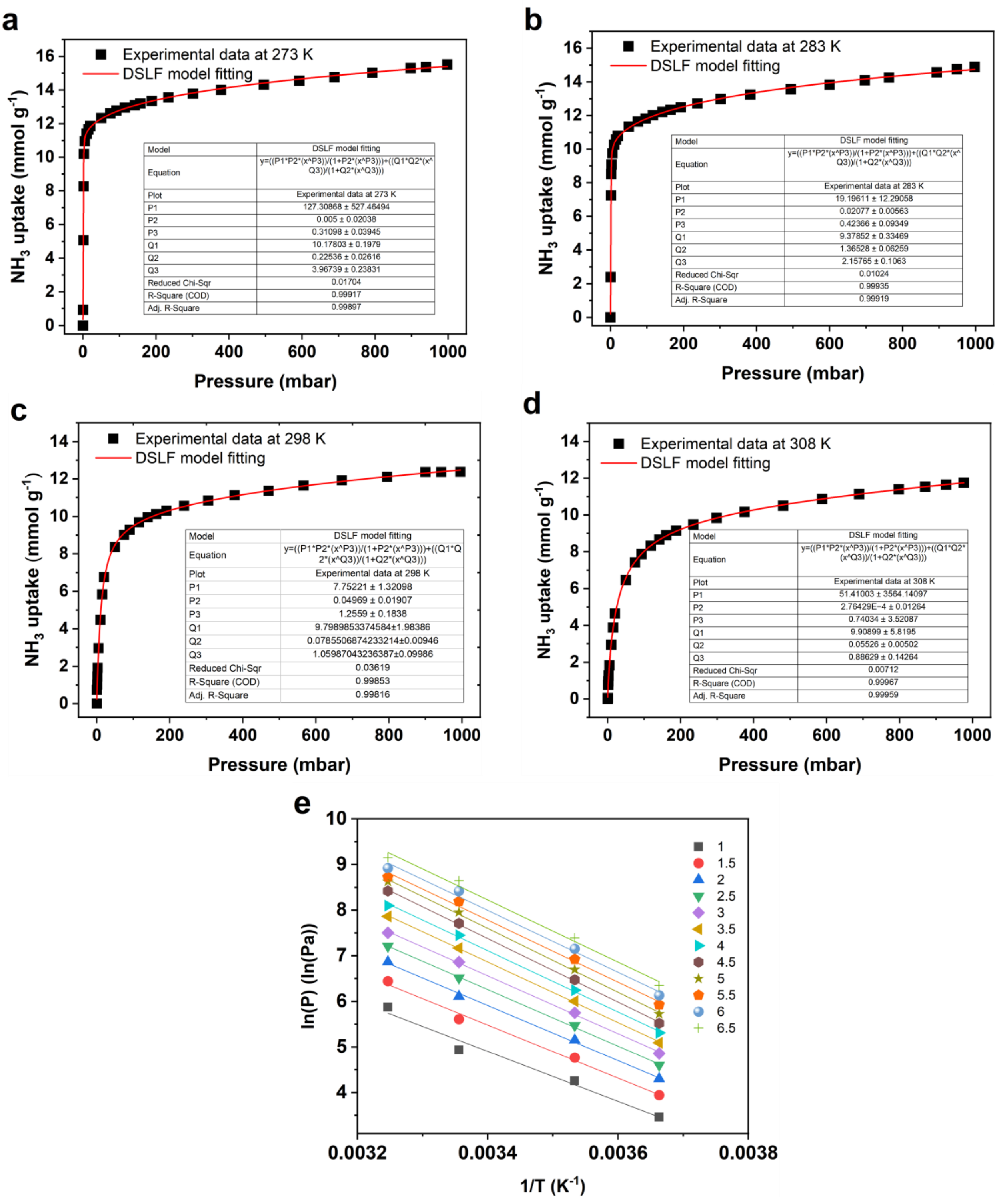

Supplementary Figure 7. Analysis and derivation of the isosteric heats of adsorption. (a-d) Fitting of isotherm by Dual-Site Langmuir Freundlich (DSLF) model for NH_3-loaded MIL-160 at 273, 283, 298 and 308 K up to 1.0 bar. (e) van't Hoff linear fittings.

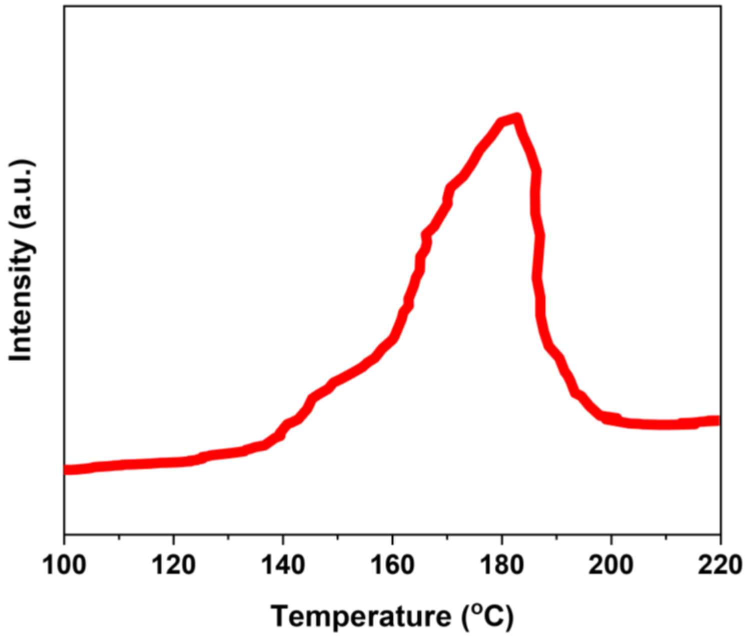

Supplementary Figure 8. Ammonia temperature-programmed desorption (TPD). NH_3-temperature programmed desorption (TPD) curve for MIL-160.

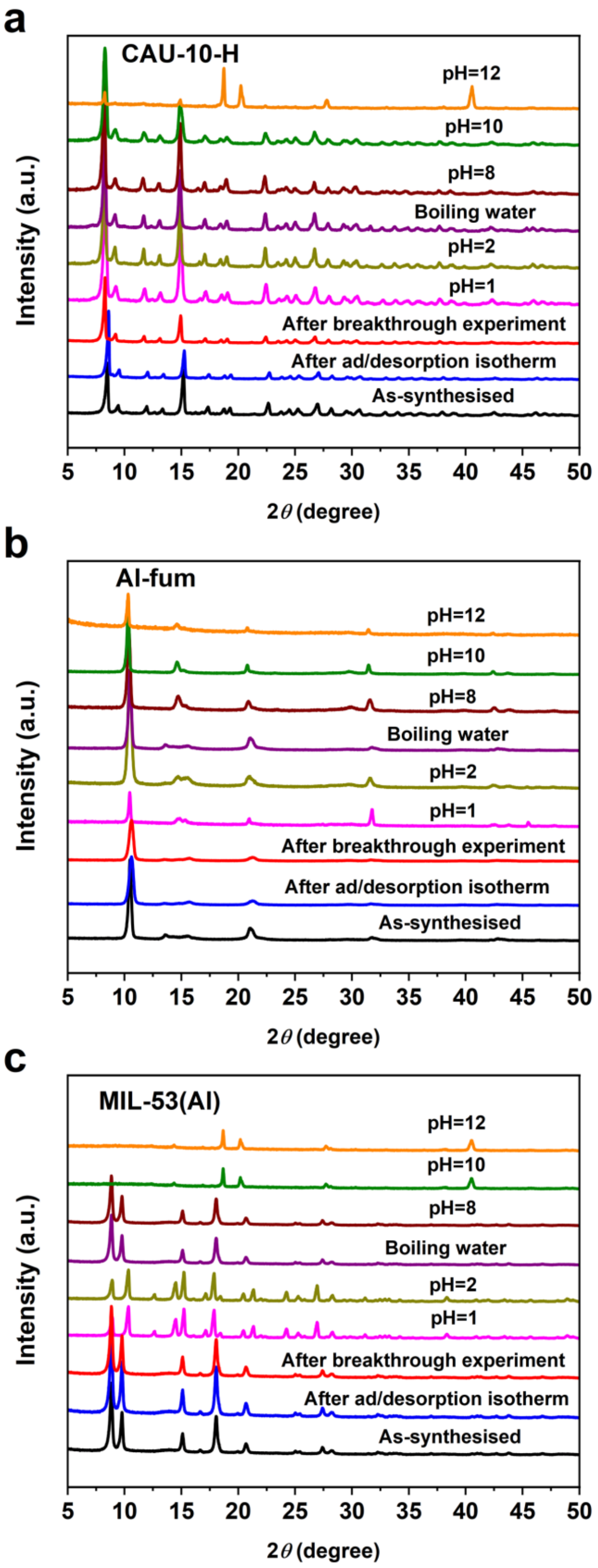

Supplementary Figure 9. Stability test. PXRD patterns of (a) CAU-10-H, (b) Al-fum and (c) MIL-53 (Al) for as-synthesised samples and samples after NH_3 isotherms and dynamic breakthrough experiments and samples after soaked in solutions with pH=1, 2, 8, 10, 12 and in boiling water for 12 h.

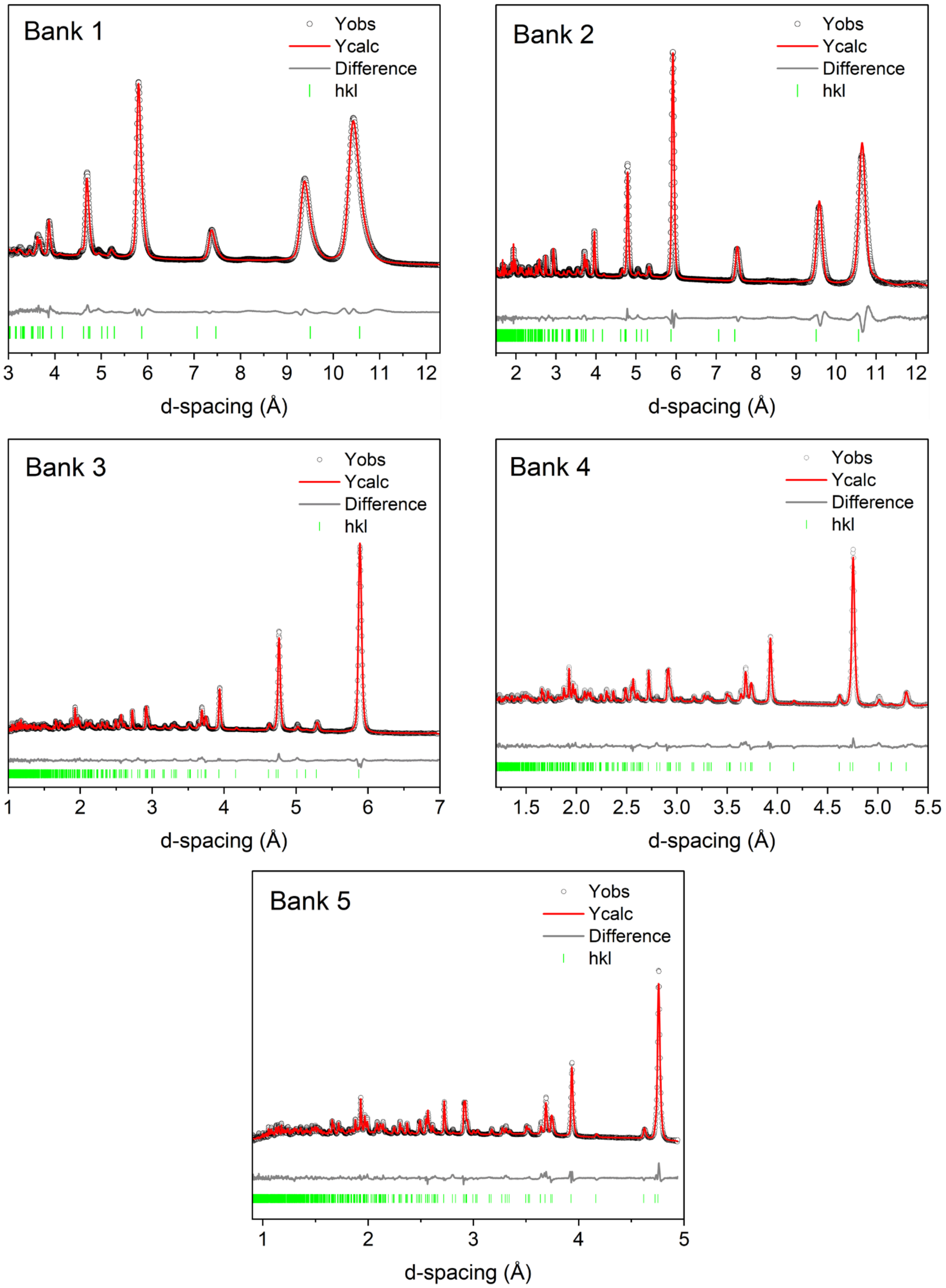

Supplementary Figure 10. Neutron powder diffraction. Rietveld refinement patterns of the NPD data of bare MIL-160 from bank 1 to 5.

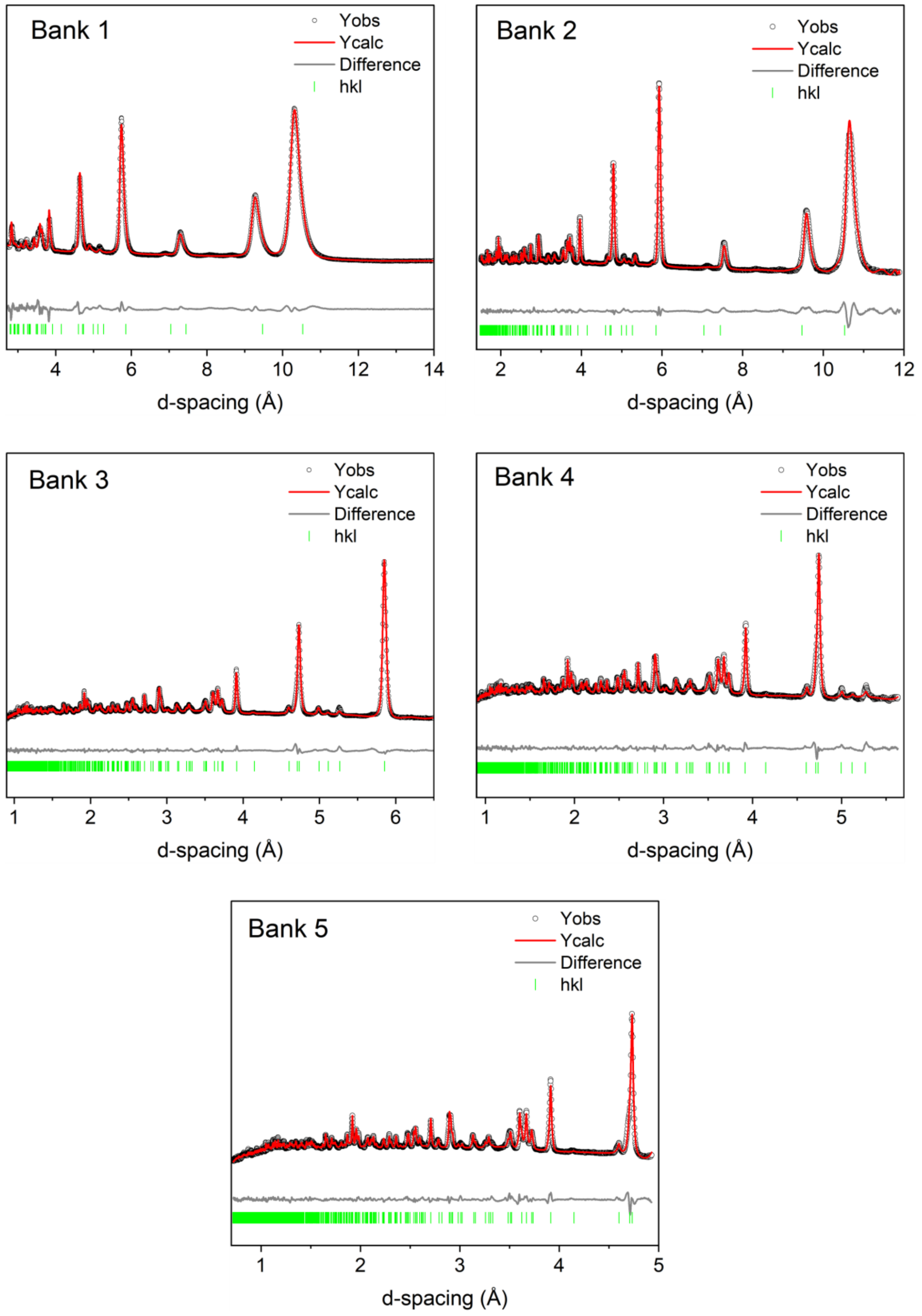

Supplementary Figure 11. Neutron powder diffraction. Rietveld refinement patterns of the NPD data of MIL-160·$(ND_3)_{0.4}$ from bank 1 to 5. Due to excessivly dosed ND_3 and rapid cooling, trace amount of solid ammonia with cubic structure[2] was identified through Pawley refinement and related peaks were treated as anomalous background in the Rietveld refinement.

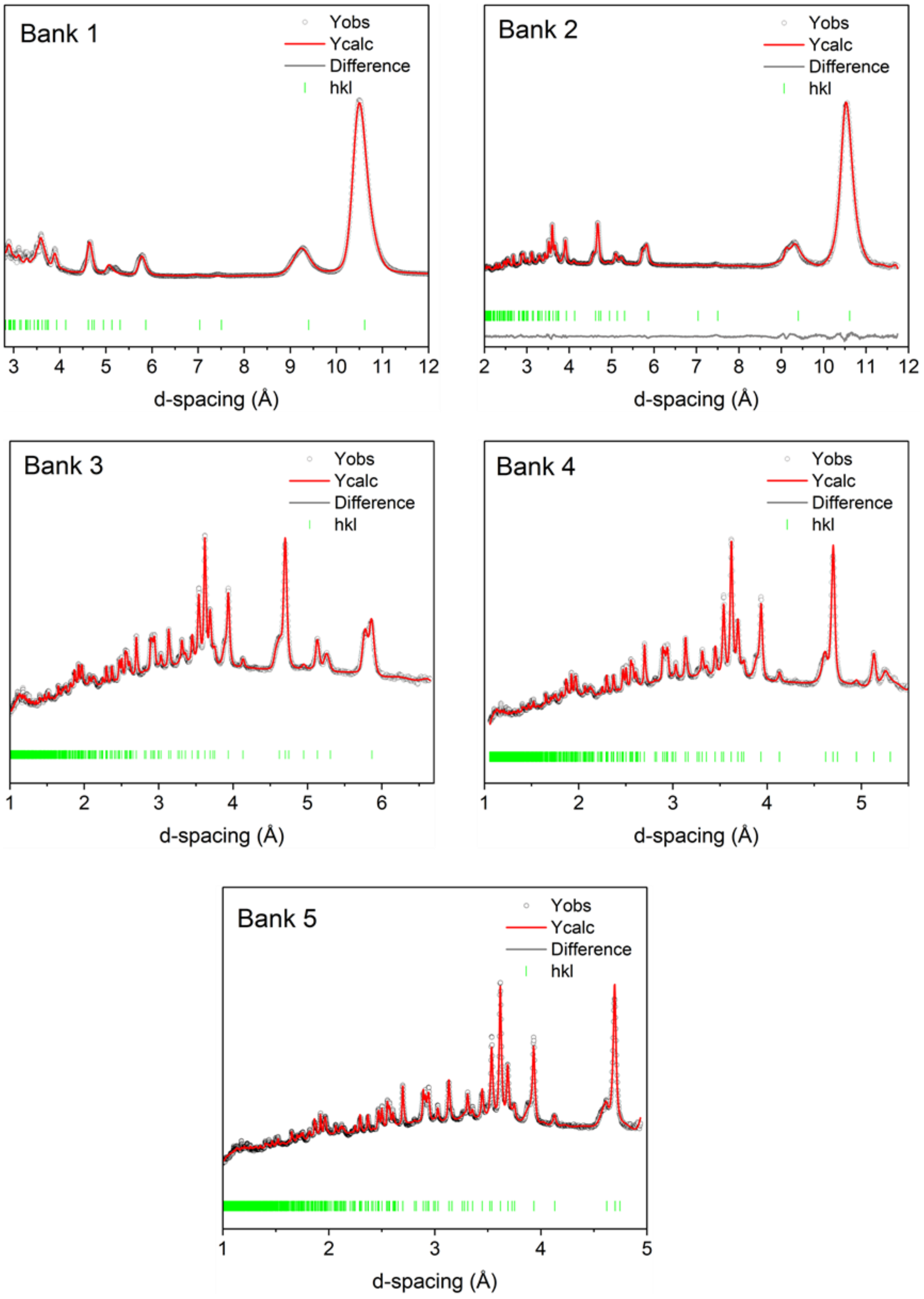

Supplementary Figure 12. Neutron powder diffraction. Rietveld fit profiles of MIL-160· $(ND_3)_{1.5}$ from bank 1 to 5. Due to excessively dosed ND_3 and presence of trace moisture in the pipeline, a series of ammonia monohydrate[3-6] was identified via Pawley refinement and related peaks were treated as anomalous background in Rietveld refinement.

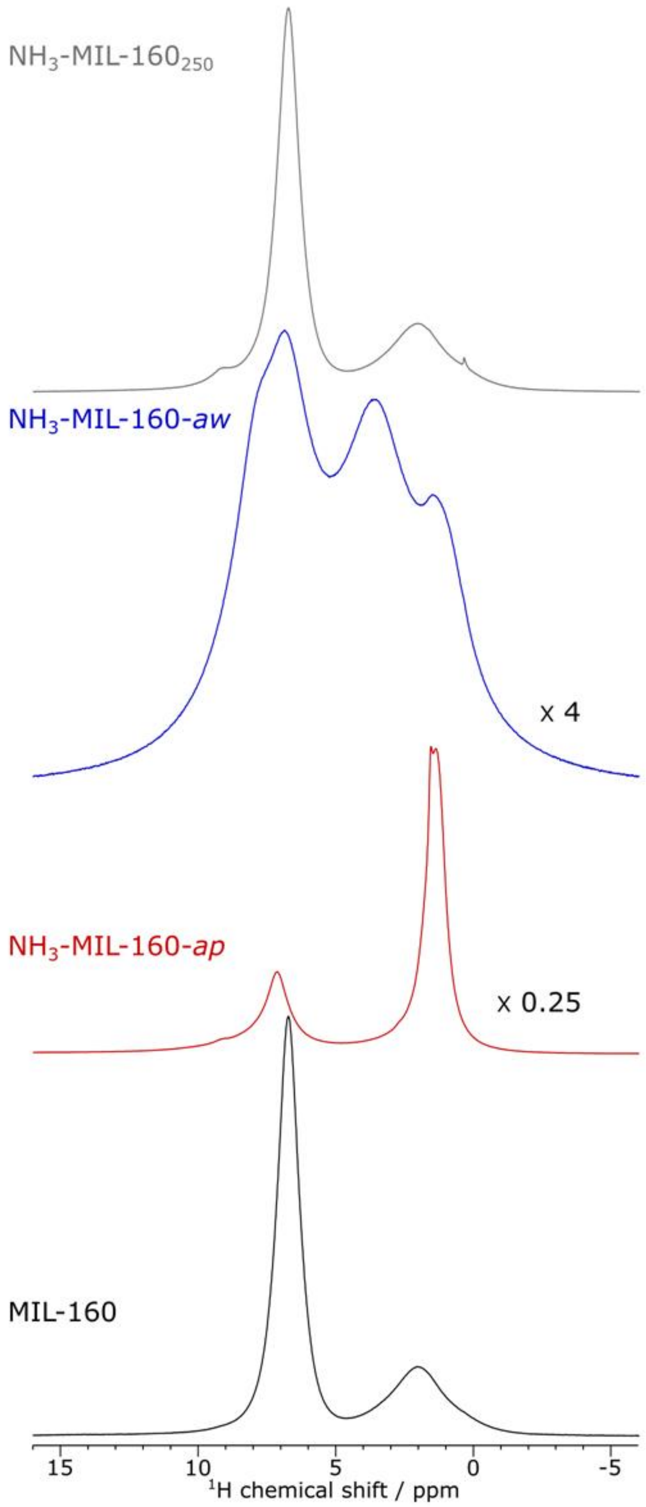

Supplementary Figure 13. ^{1}H Hahn-echo MAS NMR spectra of the MIL-160 samples. 1 rotor period was used as the delay either side of the π-pulse for the Hahn-echo. MIL-160 is pristine sample (black); NH_3-MIL-160-*ap* (red) is the MIL-160 sample as-prepared after NH_3 dosing; NH_3-MIL-160-*aw* (blue) is this dosed sample after one week of ambient storage; and NH_3-MIL-160_{250} (gray) is this latter sample after heating to 250 °C.

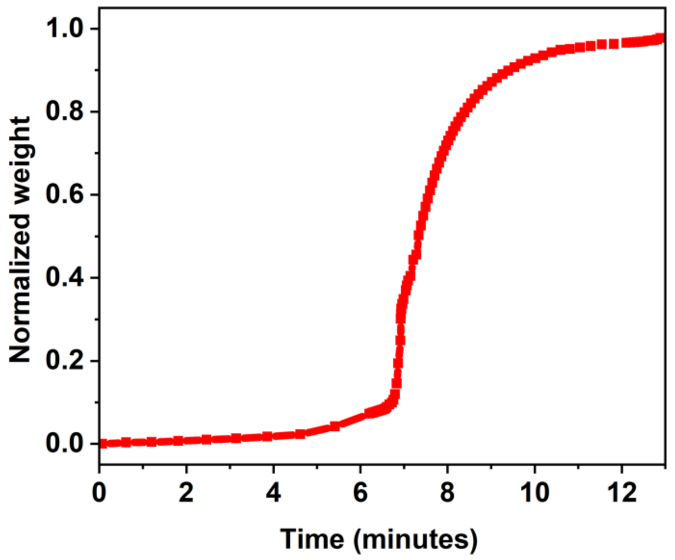

Supplementary Figure 14. Kinetic analysis. Adsorption kinetics of NH_3 in MIL-160 from 1.9 to 3.0 mbar at 298 K.

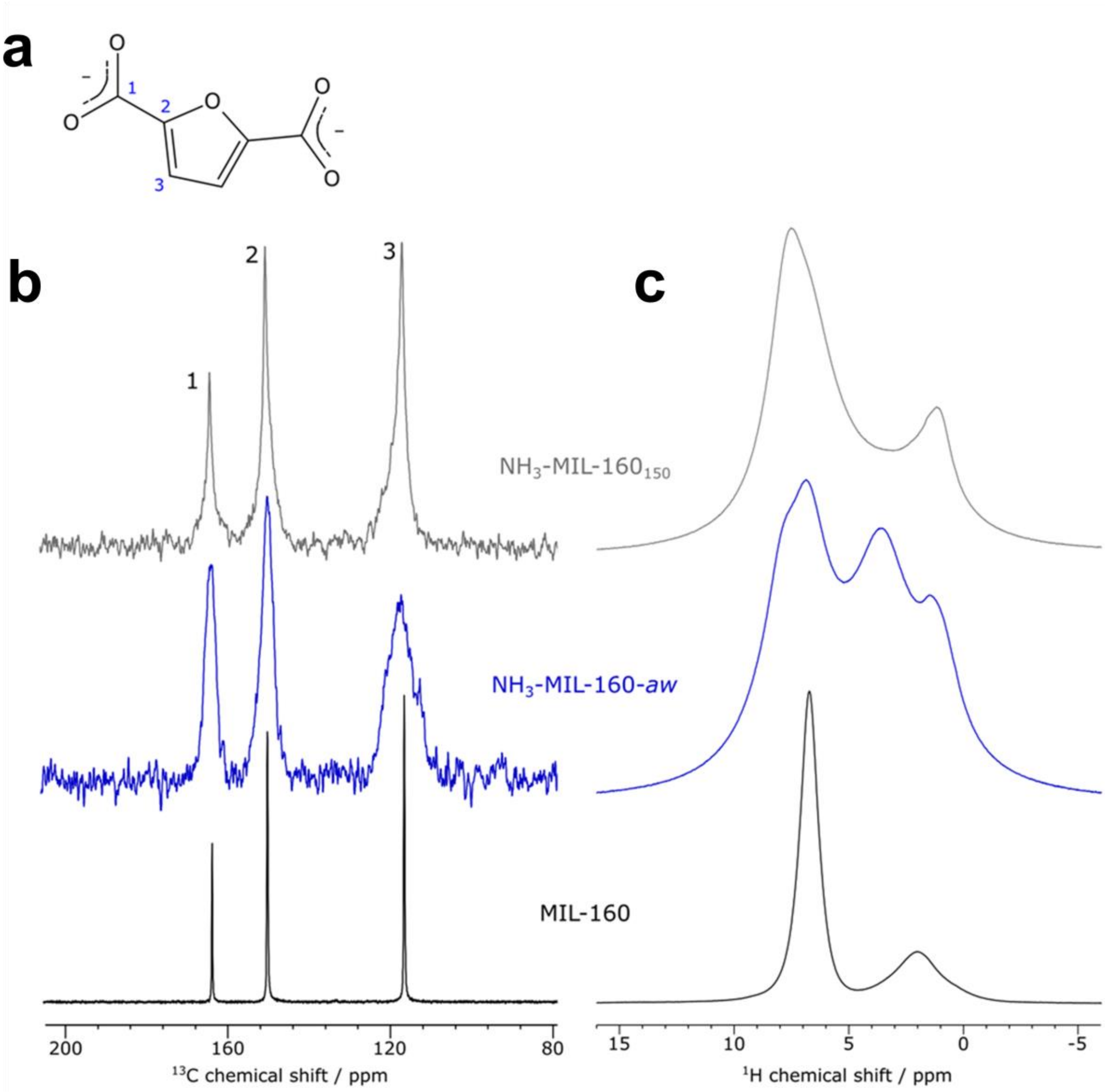

Supplementary Figure 15. ssNMR spectrum. (a) ligand structure of MIL-160, (b) {^{1}H-}^{13}C CPMAS and (c) ^{1}H Hahn-echo MAS NMR spectra of select MIL-160 samples. 1 rotor period was used as the delay either side of the π-pulse for the Hahn-echo. The ^{1}H Hahn-echo MAS NMR spectra of MIL-160 (black) and NH_3-MIL-160-*aw* (blue) are reproduced from Supplementary Figure 14 for comparison. After loading with NH_3, subsequent desorption at 150 °C, NH_3-MIL-160_{150} (gray) does not fully regenerate the MOF structure.

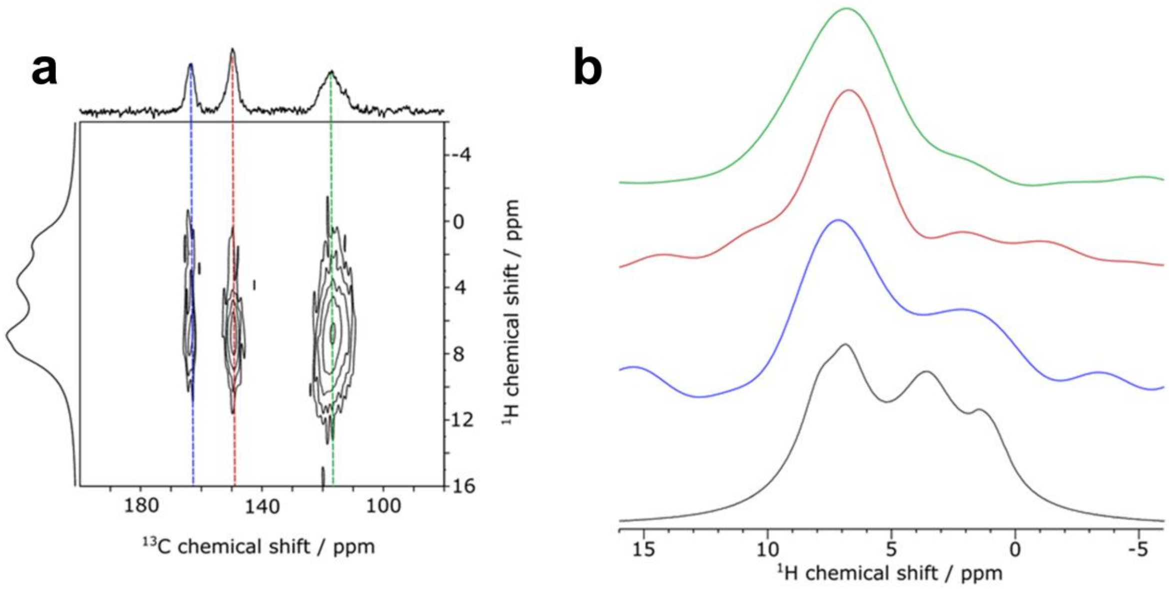

Supplementary Figure 16. ssNMR spectrum. (a) ^{1}H-^{13}C heteronuclear dipolar correlation spectrum of NH_3-MIL-160-*aw* and (b) corresponding cross-sectional slices taken at the positions indicated by the coloured dashed lines. The ^{1}H Hahn-echo MAS NMR spectrum for this sample is also reproduced (right, black) for comparison.

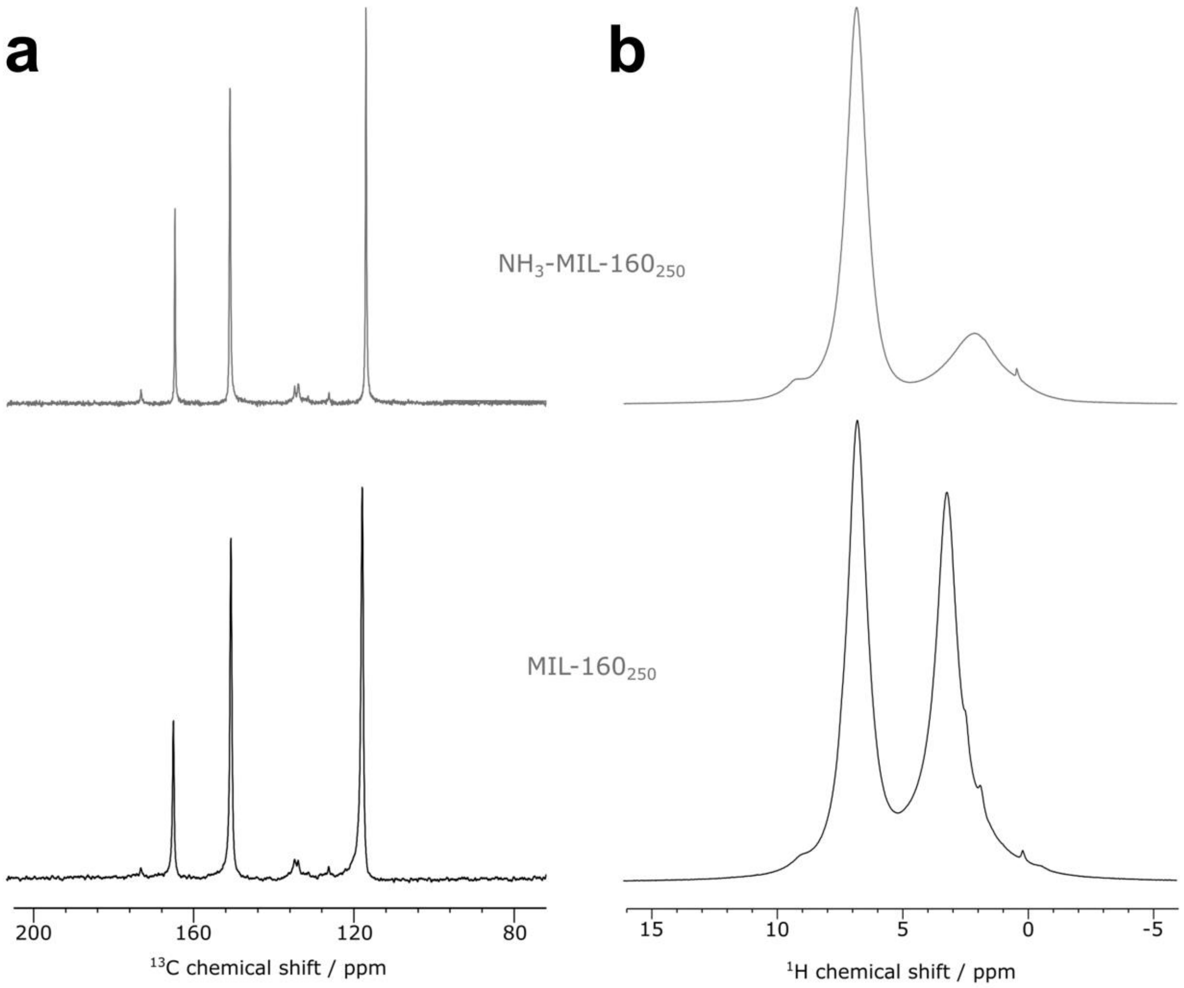

Supplementary Figure 17. ssNMR spectrum. (a) Comparison of {^{1}H-}^{13}C CPMAS and (b) ^{1}H Hahn-echo MAS NMR spectra of NH_3 loaded and desorbed at 250 ºC for MIL-160 (gray) and activated pristine MIL-160 that has undergone heating at 250 ºC (black). 1 rotor period was used as the delay either side of the π-pulse for the Hahn-echo.

Supplementary Notes

Supplementary Note 1. Analysis and Derivation of the Isosteric Heats of Adsorption

The isosteric enthalpies (ΔH_n) and entropies of adsorption (ΔS_n) were calculated as a function of NH_3 (n) from the isotherms that were measured over a range of temperatures (273–308 K) using the Clausius−Clapyeron Supplementary equation (1).

$$\ln(P)_n = \frac{\Delta H_n}{RT} - \frac{\Delta S_n}{R} \qquad \text{Supplementary equation (1)}$$

where p is pressure in Pa, T is the temperature, and R is the ideal gas constant. A graph of ln*(p)* versus $1/T$ at constant loading allows the differential enthalpy and entropy of adsorption and the isosteric enthalpy of adsorption (Q_{st}, n) to be determined.

Supplementary Note 2. Solid-state Nuclear Magnetic Resonance Spectroscopy

Solid-state NMR spectra were recorded using a Bruker 9.4 T (400 MHz ^{1}H Larmor frequency) AVANCE III spectrometer equipped with a 4 mm HFX MAS probe. Experiments were acquired at ambient temperature using a MAS frequency of 12 kHz and the number of scans varied from 16 (for ^{1}H NMR) through 128 (for ^{27}Al NMR) to 2048 (for {^{1}H-}^{13}C cross-polarisation (CP)). ^{1}H pulses of 100 kHz were used for all experiments and for SPINAL-64 heteronuclear decoupling[7] during ^{13}C and ^{27}Al acquisition.

For the ^{27}Al direct excitation experiments a hard ($\nu_{rf} \approx 70$ kHz) ^{27}Al 0.5 μs pulse was used. For the {^{1}H-}^{13}C CPMAS experiments, 2 ms CP mixing time was employed using a ramped (70-100%) transfer pulse on the ^{1}H channel with 73 kHz maximum amplitude to match a square ^{13}C spin-lock pulse of 50 kHz.

Samples were packed into 4.0 mm o.d. zirconia rotors, treated, and then sealed with a Kel-F rotor cap. The treatments included activation (10 hours at 150 ºC under dynamic vacuum) [MIL-160], as-prepared partial loading with NH_3 (30 mins) [NH_3-MIL-160-*ap*], equilibration with NH_3 (after 1 week in ambient storage) [NH_3-MIL-160-*aw*], desorption at 150 ºC (10 hours under dynamic vacuum) [NH_3-MIL-160_{150}], desorption at 250 ºC (10 hours under dynamic vacuum) [NH_3-MIL-160_{250}], and activated pristine MIL-160 heated to 250 ºC (10 hours under dynamic vacuum) [MIL-160_{250}].

Spectral simulations were performed in the solid lineshape analysis (SOLA) module v2.2.4 in Bruker TopSpin v4.0.9 and using Dmfit[8] for a Gaussian Isotropic Model to represent the distribution in isotropic chemical shift and quadrupolar parameters. The ^{1}H and ^{13}C NMR chemical shifts were referenced to neat TMS externally, and the ^{27}Al chemical shifts were referenced externally to 1.1 mol/kg $Al(NO_3)_3$ in D_2O.

Supplementary Tables

Supplementary Table 1. Summary of the dynamic dry NH_3 breakthrough capacities of selected stable MOF materials.

MOF materials	Measure condition	NH_3 dynamic capacity (mmol g^{-1})	Reference
MIL-160	1000 ppm, 298 K	4.2	**This work**
CAU-10-H	1000 ppm, 298 K	1.3	**This work**
Al-fum	1000 ppm, 298 K	0.4	**This work**
MIL-53(Al)	1000 ppm, 298 K	0.15	**This work**
UiO-66-X (M=defect, Cu^{I}, Cu^{II})	630 ppm, 298 K	2.07, 3.07, 4.15	9
MFM-303(Al)	833 ppm, 298 K	2.9	10
Mg_2(dobpdc)	570 ppm, 298 K	8.25	11
MFM-300(M) (M=Cr, V^{III}, V^{IV}, Fe)	1000 ppm, 298 K	1.1, 1.9, 1.0, 0.6	12
MFM-300(Sc)	1000 ppm, 298 K	1.65	13
Co_2Cl_2BTDD	1000 ppm, 293 K	4.78	14

Supplementary Table 2. Summary of the enthalpy of adsorption of selected MOF materials.

MOF materials	Structure	Q_{st} (kJ mol^{-1})	Reference
MIL-160	μ_2-OH	45−63	**This work**
MFM-300(Fe)	μ_2-OH	35−40	12
MFM-300(Al)	μ_2-OH	30−50	15
MFM-300(Cr)	μ_2-OH	40−65	12
MFM-300(Sc)	μ_2-OH	30−60	13
UiO-66-defect	μ_3-OH	15−40	9
UiO-66-Cu^{I}	μ_3-OH and open metal sites	5−40	9
UiO-66-Cu^{II}	μ_3-OH and open metal sites	25−55	9
MFM-303(Al)	μ_2-OH and −COOH	61.5	10
$Ni_2(adc)_2$(dabco)	open metal sites	18	16
Mn_2Cl_2(BTDD)	open metal sites	20-45	14
Co_2Cl_2(BTDD)	open metal sites	35-75	14
Ni_2Cl_2BTDD	open metal sites	50-120	14

Supplementary Table 3. Summary of the pore volume, NH_3 isothermal adsorption capacities and NH_3 packing density in stable MOF materials.

MOF	Measuring condition	Pore volume ($cm^3\ g^{-1}$)	NH_3 isothermal uptake ($mmol\ g^{-1}$)	NH_3 packing density/$g\ cm^{-3}$	Reference
**MIL-160**	**298 K 1.0 bar**	**0.45**[a]	**12.8**	**0.48**	**This work**
	**273 K 1.0 bar**		**15.5**	**0.59**	
**CAU-10-H**	**298 K 1.0 bar**	**0.32**[a]	**10.0**	**0.53**	**This work**
**Al-fum**	**298 K 1.0 bar**	**0.44**[a]	**8.9**	**0.34**	**This work**
**MIL-53(Al)**	**298 K 1.0 bar**	**0.31**[a]	**3.0**	**0.16**	**This work**
MOF-303	298 K 1.0 bar	0.55[a]	19.7	0.61 (293 K)	17
MFM-303(Al)	293 K 1.0 bar	0.191[b]	9.0	0.80	10
UiO-66-X (M=defect, Cu^{I}, Cu^{II})	273 K 1.0 bar	0.388[b]	11.8, 12.6, 16.9	0.52, 0.55, 0.74	2
MFM-300(M) (M=V^{III}, V^{IV}, Fe)	273 K 1.0 bar	0.49, 0.48, 0.46[a]	16.1, 17.3, 15.6	0.54, 0.61, 0.60	12
MFM-300(Sc)	298 K 1.0 bar	0.48[a]	13.1	0.46	13
MFM-300(Al)	273 K 1.0 bar	0.37[c]	15.7	0.72	8
Ni_acryl_TMA	298 K 1.0 bar	0.57[a]	23.5	0.70	18
Ni_acryl_TGA	298 K 1.0 bar	0.56[a]	17.4	0.53	18
M_2Cl_2BTDD (M=Mn, Co, Ni)	298 K 1.0 bar	n/a	15.5, 12.0, 12.0	n/a	14
M_2(dobpdc) (M=Mn, Co, Ni, Mg)	298 K 1.0 bar	1.18, 1.06, 1.11, 1.60[a]	13.3, 13.3, 20.8, 23.9	0.19, 0.21, 0.32, 0.25	11
$M_2(adc)_2$(dabco) (M=Zn, Co, Ni)	295 K 1.0 bar	0.25[a]	8.3, 11.2, 12.1	0.56, 0.76, 0.82	16
NU-1401	298 K 1.0 bar	0.23[a]	8.4	0.62	19
Al-PMOF	298 K 1.0 bar	n/a	7.7	n/a	20

[a]: pore volume determined from N_2 isotherms at 77 K.

[b]: pore volume from crystal structure.

[c]: pore volume determined from CO_2 isotherms at 273 K.

Supplementary Table 4. Crystal Data and Details of the Structure Determination for ND_3 loaded MIL-160.

	MIL-160	MIL-160·$(ND_3)_{0.4}$	MIL-160·$(ND_3)_{1.5}$
Formula	[Al(OH)fdc], $C_6H_3AlO_6$	[Al(OH)fdc]·$(ND_3)_{0.4}$, $C_6H_3D_{1.2}AlO_6N_{0.4}$	$[Al(OH)_{0.176}(OD)_{0.824}fdc]$ ·$(ND_3)_{1.228}$·$(NH_3)_{0.274}$, $C_6H_3D_{4.5}AlO_6N_{1.5}$
Formula weight	198.1	206.1	223.5
Crystal system	Tetragonal	Tetragonal	Tetragonal
Space Group	$I4_1/amd$ (141)	$I4_1/amd$ (141)	$I4_1/amd$ (141)
a, *b* (Å)	21.1298(1)	21.0660(12)	21.224(2)
c (Å)	10.6406(5)	10.6056(6)	10.4837(11)
Volume(Å^3)	4750.7(5)	4706.5(6)	4722.3(12)
ρ (calc) g/cm^3	1.10770	1.166	1.283
Radiation type	Neutron	Neutron	Neutron
Scan method	Time of flight	Time of flight	Time of flight
R_{exp} (%)	0.323	0.321	0.281
R_{wp} (%)	1.854	1.710	0.957
R_p (%)	1.514	1.498	0.821
GoF (χ^2)	5.739	5.329	3.401
CCDC	2219217	2219215	2219216

Supplementary Table 5. Host–Guest Interactions in MIL-160·$(ND_3)_{0.4}$.

MIL-160·0.5(ND_3)	Interactions	Distances (Å)	Colour
Site I	H (HO−Al)···N (site I)	2.36(2)	Blue
	D (site I)···C=C	2.99(4)	Red
	N (site I)···H−C	2.11(2)	Aqua
	D (site I)···O_{ligand}	2.20(1)	Dark yellow
	N (site I)···N (site II)	2.82(3)	Violet
Site II	D (site II)···O_{ligand}	3.17(2)	Rose
	N(site II)···N (site I)	2.82(3)	Violet

Supplementary Table 6. Atomic positions for atoms in MIL-160·$(ND_3)_{0.4}$.

	x	y	z	Occupancy	Biso / Å^2
Al	0.3197(4)	0	0	1	1.3(3)
O1	0.25749(19)	0.00749(19)	0.125	1	3.00(9)
H1	0.2266(5)	0.0234(5)	0.125	1	3.60(10)
O2	0.3824(5)	0.0068(3)	0.1217(9)	1	3.00(9)
O3	0.3375(3)	0.0656(4)	0.2790(12)	1	3.00(9)
H3	0.4341(5)	0.1047(3)	0.4570(9)	1	2.36(8)
C1	0.3868(3)	0.0435(2)	0.2199(8)	1	1.96(7)
C2	0.45080(18)	0.05676(12)	0.2713(3)	1	1.96(7)
C3	0.4653(3)	0.0854(2)	0.3825(5)	1	1.96(7)
O4	0.5	0.04076(6)	0.20924(6)	1	3.00(9)
N1	0.4658(19)	0.3648(14)	0.811(3)	0.110(4)	1.0(12)
D1	0.484(6)	0.397(3)	0.872(7)	0.110(4)	1.2(15)
D2	0.486(5)	0.322(3)	0.834(8)	0.110(4)	1.2(15)
D3	0.484(4)	0.376(5)	0.724(5)	0.110(4)	1.2(15)
N2	0.8469(9)	0.0893(8)	0.7698(18)	0.1010(19)	4.6(8)
D1	0.848(3)	0.0546(17)	0.837(4)	0.1010(19)	5.5(10)
D2	0.8186(19)	0.1242(16)	0.805(4)	0.1010(19)	5.5(10)
D3	0.8226(17)	0.070(3)	0.696(3)	0.1010(19)	5.5(10)

Supplementary Table 7. Host–Guest Interactions in MIL-160·$(ND_3)_{1.5}$.

MIL-160·0.5(ND_3)	Interactions	Distances (Å)	Colour
Site I	H (HO−Al)···N (site I)	2.31(2)	Blue
	D (site I)···O_{ligand}	2.32(4)	Dark yellow
	N (site I)···H−C	2.53(2)	Turquiose
	D (site I)···C=C	3.60(4)	Red
	N (site I)···N (site II)	4.11(2)	Lime
	N (site I)···N (site III)	3.79(4)	Violet
Site II	D (site II)···O_{ligand}	2.94(2)	Rose
	N (site II)···N (site I)	4.11(2)	Lime
Site III	N (site III)··· N (site I)	3.79(4)	Violet

Supplementary Table 8. Atomic positions for atoms in MIL-160·$(ND_3)_{1.5}$.

	x	y	z	Occupancy	Biso / Å^2
Al	0.3216(9)	0	0	1	4.3(6)
O1	0.2676(5)	0.0176(5)	0.125	1	6.6(2)
H1	0.2334(7)	0.0166(7)	0.125	0.176(14)	8.0(3)
D1	0.2334(7)	0.0166(7)	0.125	0.824(14)	8.0(3)
O2	0.3821(9)	0.0086(6)	0.1287(17)	1	6.6(2)
O3	0.3385(6)	0.0700(8)	0.273(2)	1	6.6(2)
H3	0.4354(9)	0.1200(6)	0.4494(17)	1	6.37(19)
C1	0.3863(5)	0.0472(5)	0.2192(15)	1	5.31(16)
C2	0.4495(3)	0.0630(2)	0.2693(6)	1	5.31(16)
C3	0.4669(6)	0.0968(4)	0.3762(10)	1	5.31(16)
O4	0.5	0.04322(10)	0.20678(10)	1	6.6(2)
N1	0.5	0.322(2)	0.675(5)	0.188(8)	1.3(18)
D1	0.502(9)	0.307(7)	0.767(7)	0.094(4)	2(2)
D2	0.493(7)	0.369(3)	0.680(15)	0.094(4)	2(2)
D3	0.460(3)	0.303(7)	0.639(15)	0.094(4)	2(2)
N2	0.509(3)	0.3861(6)	0.0865(13)	0.179(3)	7.7(7)
D1	0.541(6)	0.417(5)	0.125(3)	0.179(3)	9.3(8)

D2	0.489(4)	0.363(2)	0.162(2)	0.179(3)	9.3(8)
D3	0.474(4)	0.413(5)	0.049(4)	0.179(3)	9.3(8)
H1	0.1114(8)	0.421(3)	0.864(3)	0.137(2)	8.0(4)
H2	0.1848(11)	0.4267(16)	0.907(2)	0.137(2)	8.0(4)
H3	0.166(2)	0.4338(14)	0.7576(18)	0.137(2)	8.0(4)
N1	0.1559(5)	0.4088(5)	0.8383(11)	0.478(3)	6.7(4)
D1	0.1114(8)	0.421(3)	0.864(3)	0.341(4)	8.0(4)
D2	0.1848(11)	0.4267(16)	0.907(2)	0.341(4)	8.0(4)
D3	0.166(2)	0.4338(14)	0.7576(18)	0.341(4)	8.0(4)

Supplementary References

1. Cadiau, A., Lee, J. S., Borges, D. D., Fabry, P., Devic, T., Wharmby, M. T., Martineau, C., Foucher, D., Taulelle, F., Jun, C.H., Hwang, Y. K., Stock, N., Lange, M.F.D., Kapteijn, F., Gascon, J., Maurin, G., Chang, J. S., Serre, C. Design of hydrophilic metal-organic framework water adsorbents for heat reallocation. *Adv. Mater.* **27**, 4775–4780 (2015).
2. Olovsson, I., Templeton, D. H. X-ray study of solid ammonia. *Acta Cryst.* **12**, 832–836 (1959).
3. Olovsson, I., Templeton, D. H. The crystal structure of ammonia monohydrate. *Acta Cryst.* **12**, 827–832 (1959).
4. Fortes, A. D., Suard, E., Cailleau, M. H. L., Pickard, C. J., Needs, R. J. Crystal structure of ammonia monohydrate phase II. *J. Am. Chem. Soc.* **131**, 13508–13515 (2009).
5. Wilson, C. W., Bull, C. L., Stinton, G., Loveday, J. S. Pressure-induced dehydration and the structure of ammonia hemihydrate-II. *J. Chem. Phys.* **136**, 094506 (2012).
6. Loveday, J. S., Nelmes, R. J., Marshall, W. G., Besson, J. M., Klotz, S., Hamel, G. Structure of deuterated ammonia IV. *Phys. Rev. Lett.* **76**, 74 (1996).
7. Fung, B.M., Khitrin, A.K., Ermolaev, K., An improved broadband decoupling sequence for liquid crystals and solids. *J. Magn. Reson.* **142**, 97–101 (2000).
8. Massiot, D., Fayon, F., Capron, M., King, I., Calv´e, S. L., Alonso, B., Durand, J., O., Bujoli, B., Gan, Z. H., Hoatson, G. Modelling one- and two-dimensional solid-state NMR spectra. *Magn. Reson. Chem.* **40**, 70–76 (2002).
9. Ma, Y., Lu, W., Han, X., Chen, Y., Silva, I., Lee, D., Sheveleva, A., Wang, Z., Li, J., Li, W., Fan, M., Xu, S., Tuna, F., McInnes, E., Cheng, Y., Rudic, S., Manuel, P., Frogley, M., Cuesta, A., Schroder, M., Yang, S. Direct observation of ammonia storage in UiO-66 incorporating Cu(II) binding sites. *J. Am. Chem. Soc.* **144**, 8624–8632 (2022).
10. Marsh, C., Han, X., Li, J., Lu, Z., Argent, S. P., da Silva, I., Cheng, Y., Daemen, L. L., Ramirez-Cuesta, A. J., Thompson, S. P., Blake, A. J., Yang, S., Schroder, M. Exceptional packing density of ammonia in a dual-functionalized metal-organic framework. *J. Am. Chem. Soc.* **143**, 6586−6592 (2021).
11. Kim, D. W., Kang, D. W., Kang, M., Lee, J. H., Choe, J. H., Chae, Y. S., Choi, D. S., Yun, H., Hong, C. S. High ammonia uptake of a metal-organic framework adsorbent in a wide pressure range. *Angew. Chem. Int. Ed.* **59**, 22531−22536 (2020).
12. Han, X., Lu, W., Chen, Y., da Silva, I., Li, J., Lin, L., Li, W., Sheveleva, A. M., Godfrey, H. G. W., Lu, Z., Tuna, F., McInnes, E. J. L., Cheng, Y., Daemen, L. L., McPherson, L. J. M., Teat, S. J., Frogley, M. D., Rudic, S., Manuel, P., Ramirez-Cuesta, A. J., Yang, S., Schroder, M. High ammonia adsorption in MFM-300 materials: dynamics and charge transfer in host-guest binding. *J. Am. Chem. Soc.* **143**, 3153−3161 (2021).
13. Guo, L., Han, X., Ma, Y., Li, J., Lu, W., Li, W., Lee, D., Silva, I., Cheng, Y., Rudic, S., Manuel, P., Frogley, M., Cuesta, A., Schroder, M., Yang, S. High capacity ammonia adsorption in a robust metal–

organic framework mediated by reversible host–guest interactions. *Chem. Commun.*, **58**, 5753−5756 (2022).

14. Rieth, A. J., Tulchinsky, Y., Dincă, M. High and reversible ammonia uptake in mesoporous azolate metal–organic frameworks with open Mn, Co, and Ni sites. *J. Am. Chem. Soc*. **138**, 9401–9404(2016).
15. Godfrey, H. G. W., da Silva, I., Briggs, L., Carter, J. H., Morris, C. G., Savage, M., Easun, T. L., Manuel, P., Murray, C. A., Tang, C. C., Frogley, M. D., Cinque, G., Yang, S., Schroder, M. Ammonia storage by reversible host−guest site exchange in a robust metal-organic framework. *Angew. Chem. Int. Ed.* **130**, 14994−14997 (2018).
16. Cao, Z., Landström, K. N., Akhtar, F. Rapid ammonia carriers for CR systems using MOFs $[M_2(adc)_2(dabco)]$ (M=Co, Ni, Cu, Zn). *Catalysts.* **10**, 1444–1454 (2020).
17. Wang, Z., Li, Z., Zhang, X. G., Xia, Q., Wang, H., Wang, C., Wang, Y., He, H., Zhao, Y., Wang, J. Tailoring multiple sites of metal-organic frameworks for highly efficient and reversible ammonia adsorption. *ACS Appl. Mater. Interfaces* **13**, 56025−56034 (2021).
18. Kim, D. W., Kang, D. W., Kang, M., Choi, D. S., Yun, H., Kim, S. Y., Lee, S. M., Lee, J. H., Hong, C. S. High gravimetric and volumetric ammonia capacities in robust metal-organic frameworks prepared via double postsynthetic modification. *J. Am. Chem. Soc.* **144**, 9672−9683 (2022).
19. Zhang, Y., Zhang, X., Chen, Z., Otake, K.,Peterson, G. W.,Chen, Y., Wang, X., Redfern, L. R., Goswami, S.; Li, P., Islamoglu, T., Wang, B., Farha, O. K. A flexible interpenetrated zirconium-based metal-organic framework with high affinity toward ammonia. *ChemSusChem*. **13**, 1710–1714 (2020).
20. Moribe, S., Chen, Z., Alayoglu, S., Syed, Z. H., Islamoglu, T., Farha, O. K. Ammonia capture within isoreticular metal−organic Frameworks with rod secondary building units. *ACS Mater. Lett.* **1**, 476–480 (2019).
