# Supplementary material for: Efficient capture and storage of ammonia in robust aluminium-based metal-organic frameworks
Source: Commun Chem. 2023 Mar 24;6:55. doi: 10.1038/s42004-023-00850-4 (PMC10039057; doi:10.1038/s42004-023-00850-4)
Supplement: Supplementary file 3 — Description of Additional Supplementary Files [file 42004_2023_850_MOESM3_ESM.pdf]

Description of Additional Supplementary Files

File name: Supplementary Data 1
Description: CIF for bare MIL-160 (CCDC 2219217)

File name: Supplementary Data 2
Description: CIF for MIL-160·(ND3)0.4 (CCDC 2219215)

File name: Supplementary Data 3
Description: CIF for MIL-160·(ND3)1.5 (CCDC 2219216)
